# Anti-ganglioside anti-idiotypic vaccination: more than molecular mimicry

**DOI:** 10.3389/fonc.2012.00170

**Published:** 2012-11-20

**Authors:** Ana M. H. Vázquez, Nely Rodrèguez-Zhurbenko, Ana M. V. López

**Affiliations:** ^1^Tumor Immunology Direction, Center of Molecular ImmunologyHabana, Cuba; ^2^Innovation Division, Center of Molecular ImmunologyHabana, Cuba

**Keywords:** idiotypic network, anti-idiotypic antibody, ganglioside, vaccine, natural antibodies

## Abstract

Surgery, chemotherapy, and radiation therapy are standard modalities for cancer treatment, but the effectiveness of these treatments has reached a plateau. Thus, other strategies are being explored to combine with the current treatment paradigms in order to reach better clinical results. One of these approaches is the active immunotherapy based on the induction of anti-tumor responses by anti-idiotypic vaccination. This approach arose from Jerne’s idiotypic network theory, which postulates that B lymphocytes forms a functional network, with a role in the establishment of the immune repertoires, in the regulation of natural antibody production and even in the establishment of natural tolerance. Due to the large potential diversity of the immunoglobulin variable regions, the idiotypes repertoire can mimic the universe of self and foreign epitopes, even those of non-protein nature, like gangliosides. Gangliosides are sialic acid-containing glycolipids that have been considered attractive targets for cancer immunotherapy, based on the qualitative and quantitative changes they suffer during malignant transformation and due to their importance for tumor biology. Although any idiotype could be able to mimic any antigen, only those related to antigens involved in functions relevant for organism homeostasis, and that in consequence has been fixed by evolution, would be able not only to mimic, but also to activate the idiotypic cascades related with the nominal antigen. The present review updates the results, failures and hopes, obtained with ganglioside mimicking anti-idiotypic antibodies and presents evidences of the existence of a natural response against gangliosides, suggesting that these glycolipids could be idiotypically relevant antigens.

## GANGLIOSIDES

Gangliosides are glycosphingolipids present in the outer leaflet of the plasma membranes, where significantly contribute to the surface properties of cells (Sorice et al., 1997). These glycolipids are involved in various cellular functions, including signal transduction (Bremer et al., 1986; Fujitani et al., 2005), regulation of cell proliferation and differentiation (Hakomori, 1970; Sakiyama et al., 1972; Critchley and Macpherson, 1973; Yogeeswaran and Hakomori, 1975), cell–cell recognition (Feizi, 1985), adhesion (Cheresh et al., 1986), and cell death (De Maria et al., 1997,^1998^; Kirschnek et al., 2000; Colell et al., 2001). Gangliosides function as the Ca^2^^+^ binding co-factor in synaptic transmission (Svennerholm, 1980), and as the co-factor of membrane adenylate cyclase (Partington and Daly, 1979). Furthermore, they interact with, or are the receptors of different bioactive molecules, such as bacterial toxins (Simpson and Rapport, 1971a,b; Van Heyningen et al., 1971; Holmgren et al., 1973,1980a; Kato and Naiki, 1976; Ledley et al., 1977), glycoprotein hormones (Lee et al., 1976,^1977^; Mullin et al., 1976; Holmgren et al., 1980b), type 1 interferon (Besancon and Ankel, 1974; Vengris et al., 1976; Ankel et al., 1980), fibronectin (Kleinman et al., 1979), lymphokines (Higgins et al., 1978; Miura et al., 1979; Poste et al., 1979), and serotonin (Woolley and Gommi, 1965).

The processes accompanying malignant transformation, like oxidative stress, hypoxia, and angiogenesis, as well as the metastasis formation, induce the expression on cell membranes of potential targets for cancer immunotherapy. It has been widely documented that the expression patterns of membrane gangliosides suffer quantitative and qualitative changes during neoplastic transformations (Brady et al., 1969; Hildebrand et al., 1972; Fishman, 1974; Keranen et al., 1976; Portoukalian et al., 1976). Normal melanocytes express predominantly GM3, while GD3 increases when these cells suffer an oncogenic process (Ravindranath et al., 1991). GD3 plays a role in the regulation of cell growth and the induction of angiogenesis by the tumor cells (Hedberg et al., 2000; Zeng et al., 2000; Fredman et al., 2003). Melanoma GD3 carries a ceramide portion with a long chain fatty acid, in contrast with the GD3 expressed in the normal tissues that carries a shorter chain fatty acid (Nudelman et al., 1982). Fucosyl GM1 is a unique structure that is found in small cell lung cancers (SCLC) with a very limited expression in normal tissues (Krug et al., 2004; Tokuda et al., 2006). GD2 is highly expressed on neuro-ectodermal tumors (Heimburg-Molinaro et al., 2011) and in sarcomas (Kailayangiri et al., 2012). This ganglioside is also a cancer stem cells marker and promotes tumorigenesis (Battula et al., 2012).

A very interesting case is the one of *N*-glycolylated (NeuGc) gangliosides, since these glycolipids are not naturally expressed in humans due to a genetic deletion in the gene that codes the CMP-*N*-acetyl hydroxylase, enzyme that catalyzes the conversion of *N*-acetyl to *N*-glycolyl sialic acid (Irie et al., 1998; Irie and Suzuki, 1998; Olson and Varki, 2003). However, both direct and indirect studies have indicated that NeuGc is overexpressed in several human tumors (Devine et al., 1991; Kawai et al., 1991; Marquina et al., 1996; Malykh et al., 2001). The most accepted theory for this phenomenon is the incorporation of NeuGc from dietary sources. Free sialic acids from the medium can be taken up into cells via pinocytosis. The content of the resulting pinocytotic vesicles and endosomes would eventually be delivered to the lysosome, where a sialic acid transporter then delivers the molecules into the cytosol (Bardor et al., 2005). Also endogenous synthesis from glycolyl-CoA is a possibility (Malykh et al., 2001). The explanation for the differential expression of these antigens (Ag) in human normal and tumor tissues is that the rapidly growing tumor tissues might be more efficient at scavenging NeuGc. Furthermore, hypoxia induces the expression of sialin, a sialic acid transporter on tumor cells, and enhances the incorporation of the non-human sialic acid from the external milieu (Yin et al., 2006).

The gangliosides are not only attractive targets due to their over-expression on tumor cells membranes but also because of their importance for tumor biology. The metastatic capacity of the cells is strongly affected by the gangliosides expressed on the cell membranes: disialogangliosides GD2 and GD3 participate in the anchoring of the melanoma and neuroblastoma metastatic cells to the extracellular matrix proteins (Cheresh et al., 1986; Fredman et al., 2003). Comparing the ganglioside pattern expressed by the primary tumor or the metastatic cells of a melanoma patient, gangliosides expression was higher in the last ones, especially GD1. There were also abundant *O*-acetylation of GM2, GD3, and GD2, which were absent in the primary tumor. GM2 is also strongly expressed in prostate metastasis (Zhang et al., 1998), where is found in the areas of tumor cell-to-tumor cell contact indicating a role in cellular interactions and adhesion (Fredman et al., 2003).

Furthermore, gangliosides actively shed from tumors are inserted into the plasmatic membrane of surrounding cells, affecting the function of lymphocytes (Miller and Esselman, 1975; Lengle et al., 1979; Whisler and Yates, 1980; Ladisch et al., 1983; Gonwa et al., 1984), monocytes (Ladisch et al., 1984), natural killer cells (Diatlovitskaia et al., 1985), and antigen-presenting cells (Caldwell et al., 2003; Bennaceur et al., 2006). Gangliosides have been found to shift the cytokine profile from Th1 toward the Th2 in affected cells (Crespo et al., 2006). Negative modulation of CD4 molecule on T lymphocytes has been described for both the *N*-acetylated (Sorice et al., 1995) and the *N*-glycolylated variants (de Leon et al., 2006) of GM3 ganglioside. Gangliosides have been reported to block the nuclear translocation of NF-κB in human monocytes and dendritic cells (Caldwell et al., 2003). GD3, isolated from the polar lipid fraction of ovarian cancer-associated ascites, was shown to be an inhibitory factor that prevents innate immune activation of natural killer T cells (Webb et al., 2012). GM2 inhibits immunoglobulin production by B cells (Kimata and Yoshida, 1996). In this way tumor released gangliosides reinforce tumor evasion by blocking the immunological surveillance.

Despite the fact that gangliosides are poorly immunogenic, due to their self and glycolipidic nature, several reports show the presence of naturally occurring antibodies, not only in cancer patients, but also in healthy individuals, suggesting that anti-ganglioside reactivity is fixed in the natural antibodies repertoire.

## NATURALLY OCCURRING ANTI-GANGLIOSIDE ANTIBODIES

Natural antibodies are considered humoral mediators of innate immunity and recognize antigens highly conserved throughout evolution (Cojocaru et al., 2009). It has been proposed that natural auto-antibodies and auto-reactive T cells in healthy individuals may be directed to a specific and limited set of self-molecules; this selective autoimmunity has been termed the immunological homunculus (Cohen, 2007). Due to the limited number of mutations in the genes encoding the variable region of these antibodies, the repertoire of these immunoglobulins is highly conserved within species (Cojocaru et al., 2009). Several authors have described their capacity to bind foreign antigens but also self and altered self-antigens, which may be or not of protein nature (Cohen, 2007). These antibodies recognize epitopes associated with pathogens, such as phosphorylcholine of Gram-positive bacteria, lipopolysaccharide (LPS) of Gram-negative bacteria, and various molecules expressed by parasites (Ochsenbein et al., 1999; Baumgarth et al., 2005). For this reason they are considered as a first, quick anti-infection barrier that helps to guarantee the survival since the very beginning of the organisms’ life. Among their targets have also been identified intracellular molecules, such as some nuclear (e.g., histones) and cytoskeleton (e.g., actin) proteins (Coutinho et al., 1995) and single-stranded DNA (Schwartz-Albiez et al., 2009). They also recognize peptides (e.g., amyloid beta peptide), plasma membrane glycoproteins (e.g., CD90; Schwartz-Albiez et al., 2009), oxidized lipids (e.g., phosphatidylcholine), and antigens expressed by apoptotic cells (Annexin IV; Chou et al., 2009; Baumgarth, 2011). Mounting evidence suggest that natural IgM antibodies, through this self-reactivity might contribute to critical innate immune functions involved in the maintenance of tissue homeostasis, like the clearance of apoptotic cells (Chou et al., 2009), reduction of atherosclerotic lesions (Hartvigsen et al., 2009; Cesena et al., 2012), and the reinforcement of mechanisms involved in the protection from the development of autoimmune disease (Werwitzke et al., 2005; Chen et al., 2009a,b; Silverman et al., 2009; Jiang et al., 2011; Stoehr et al., 2011).

Recently it has been described the existence of auto-antibodies against tumor-associated antigens, which can arise in the patients even before the symptoms become evident and that can be detected also in healthy donors (Zhang et al., 2003; Storr et al., 2006; Chapman et al., 2008). Some of these IgM isotype, germline antibodies have been isolated from cancer patients and have proved to be able not only to recognize tumor cells, but also to kill them by different mechanisms (Bohn, 1999; Hensel et al., 2001; Jakobisiak et al., 2003; Vollmers and Brandlein, 2005, 2006; Lutz et al., 2009). Many of the detected anti-tumor antibodies bind to carbohydrate repeated motifs, including sequences of sugars contained in gangliosides (Lutz and Miescher, 2008; Cojocaru et al., 2009).

Naturally occurring antibodies reacting with tumor-associated gangliosides have been detected in cancer patients but also in healthy donors. Antibodies against GM2 and GD2 were detected in the sera of both melanoma patients and healthy individuals (Watanabe et al., 1982; Tai et al., 1985). Other authors have reported the existence in healthy donors of naturally occurring antibodies with reactivity against gangliosides like GM1, GD1a, GD1b, and GT1b (Mizutamari et al., 1994; Ravindranath et al., 1997; Lardone et al., 2006). Silent auto-reactive B clones have also been identified in cancer patients, from which human monoclonal antibodies (mAb) against gangliosides were generated. The mAb GMA1 reacted with the gangliosides GD3, GM3, and GD2 from melanoma and neuroblastoma cell lines and not normal tissues (Mukerjee et al., 1998). The human monoclonal IgM antibody 7c11.e8, also generated by fusing lymph node cells isolated from a surgical specimen of malignant melanoma reacted with GM4, GM3, and GD3. In the presence of human serum the antibody initiated a strong lysis of melanoma tumor cells in complement-dependent cellular cytotoxicity (CDCC) assays (Abdel-Wahab et al., 1993).

The presence of naturally occurring antibodies that recognize NeuGc acid present in tumor-associated glycoconjugates has also been described. It has been shown that normal human serum contains high levels of NeuGc-specific antibodies, which attract complement molecules to the surface of leukemic cells expressing NeuGc, but not other normal cells (Zhu and Hurst, 2002; Tangvoranuntakul et al., 2003; Nguyen et al., 2005; Padler-Karavani et al., 2008). Ravindranath et al. (2007), examining the sera of healthy volunteers between the ages of 18 and 90, reported that anti-ganglioside antibodies occurred naturally and that their levels decline after 50 years, which could be relevant since the cancer incidence increases with age.

Anti-ganglioside antibodies have shown to have anti-tumor cytotoxic capacities. It has been reported the ability of a murine anti-GM2 to induce apoptosis through caspase activation in lymphoma, melanoma, and lung cancer cells expressing the antigen (Retter et al., 2005). The binding of an anti-GD2 antibody to the ganglioside expressed in lung cancer cells induced apoptosis by the reduction in the levels of phosphorylation of FAK and activation of mitogen-activated kinase p38. Immunoprecipitation experiments showed a physical association of GD2 with integrins, which were associated with FAK inside the membrane. Antibody binding to ganglioside caused conformational changes in this complex, inducing the transmission of intracellular signals that mediated the apoptosis (Aixinjueluo et al., 2005). It has been also proved that anti-GD2 antibodies of healthy donors have cytotoxic capacity against neuroblastoma cells (Ollert et al., 1997).

The capacity of the mAb 14F7, a murine IgG highly specific for NeuGcGM3, to induce oncotic cell death to tumor cells expressing this antigen has been reported. This antibody induced a tumor cell death that was accompanied by cellular swelling, membrane lesion formation, and cytoskeleton activation (Carr et al., 2000; Roque-Navarro et al., 2008). Another antibody specific for *N*-glycolylated gangliosides is P3 mAb. This is an IgM, germline encoded that is able to induce complement-mediated cytotoxicity to NeuGc expressing tumor cells (Vázquez et al., 1995; Carr et al., 2002). It has been reported that naturally occurring anti-NeuGc in healthy humans were able to kill human leukemic cells that were exogenously fed with NeuGc by a complement-mediated mechanism (Nguyen et al., 2005).

These evidences suggest that the evolution has fixed an innate immunity against gangliosides in the natural antibodies repertoire, which could play an important role for tumor immune surveillance. Since the natural antibodies secreting B cells arise in the neonatal period, they could be connected and regulated by anti-Id interactions, according to Jerne’s idiotypic network theory.

## THE IDIOTYPIC NETWORK THEORY

In 1974, Neils Jerne published the Idiotypic Network theory, which gave a different view of the immune system organization and the recognition of the “self.” According to classical clonal selection theory, the immune system was “antigen driven” and in the absence of an external antigen challenge the system should be passively inactive. In contrast, according to the network theory, the immune system consists of lymphocyte clones which are stimulated and regulated by the immunoglobulins produced by other clones within the network. Since a huge diversity of idiotypes (Id) is generated by random somatic rearrangements of genes, idiotype’s complementary structures can be found not only on antigens but also on antibodies of different idiotypes. In Jerne’s own words “the immune system of a single animal, after producing specific antibodies to an antigen, continues to produce antibodies to the idiotopes of the antibodies which it has itself made. The latter anti-Id antibodies likewise display new idiotypic profiles, and the immune system turns out to represent a network of idiotypic interactions” (Jerne, 1974, 1985).

This phenomena was extensively proved firstly by Kunkel and Oudin, who showed that ordinary antibody molecules that arise in an immunized animal are antigenic and induce the formation of specific anti-antibodies (Kunkel et al., 1963; Oudin and Michel, 1963). Later experiments further demonstrated that the recognition of self-idiotopes by B or T cells is an active physiological process controlling the suppression or expansion of the immune response (Eichmann and Rajewsky, 1975; Cazenave, 1977; Urbain et al., 1977; Bona et al., 1981).

This immune network is established in the neonatal period, thus this theory predicts that the immune systems has an autonomous activity, manifested by the presence of activated lymphocytes and antibody secretion, before any external immunization. This prediction was confirmed by studies on “antigen-free” animals, which contain in their spleen and peritoneal cavity activated B cells that secret IgM antibodies and T lymphocytes that perform as effector cells, help or suppress the antibody production (Hooijkaas et al., 1984; Pereira et al., 1986).

Thus, a network of idiotypically interacting immune cells is formed, that has a dynamic equilibrium between the idiotypes, anti-Id, and the normal self-constituents of our body, influencing the shaping of the B- and T cell repertoires, and controlling auto-reactive clones.

In the 1980s, there was an interesting debate between the proponents of the network paradigm and those of the clonal selection theory and several experiments were performed that provided evidences about both ideas (Cohn, 1981, 1986; Langman and Cohn, 1986; Cazenave, 1988; Behn, 2007).

The establishment of collections of antibody-producing hybridomas, derived from normal, unimmunized mice at different stages of ontogeny, provided proof for the existence of idiotypic connectivity (Holmberg et al., 1984a). Matrices of idiotypic complementarities were established, that allowed to estimate the degree of connectivity within different B cell populations (Holmberg et al., 1984a,b, 1986a,b,c; Kearney and Vakil, 1986). High levels of connectivity were observed within collections of fetal or neonatal origin (Holmberg et al., 1984b). However, within collections from the adult lymphocyte population the degree of connectivity was 10- to 100-fold lower (Holmberg et al., 1986c). These experiments suggested that high idiotypic connectivity is not an intrinsic property of any collection of IgM antibodies, but a distinctive property of part of the perinatal antibody repertoire.

Then, Varela and Coutinho formulated the concept of second-generation immune networks (Coutinho, 1989; Varela and Coutinho, 1991), which tried to combine the two competing paradigms. They adopted the view that the immune system is formed by two compartments of B and T lymphocytes: a majority of small resting cells constituting 80–85% of the total population and a set of large activated cells making up the other 15–20% (Pereira et al., 1985, 1986), this last being the predominant in the neonatal period. The specific response to a foreign antigen would be mainly caused by the activation of resting lymphocyte clones, which are only poorly connected to the network, thus forming the peripheral part of the system. The fraction of highly connected cells forms the actual network, a compartment of naturally activated lymphocytes. The immune network includes, in addition to V-regions, all other molecules of the somatic self. This pool of connected cells may be responsible for maintenance of normal network dynamics and prevention of auto-aggression.

**FIGURE 1:**
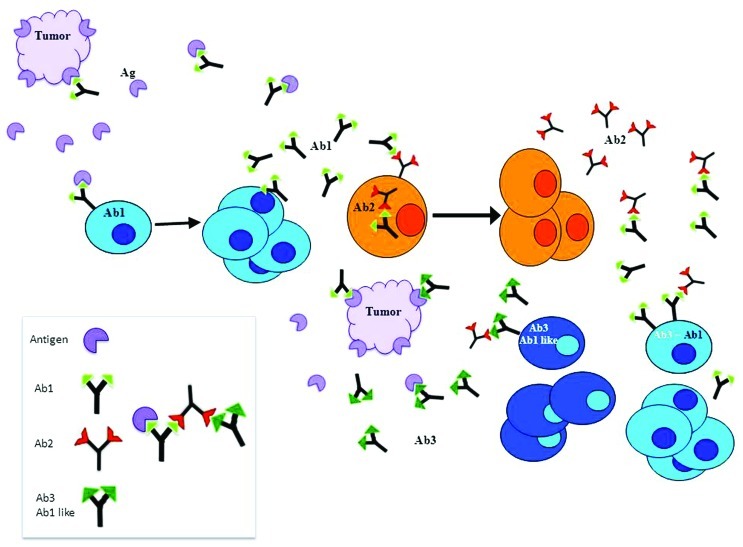
**According to the idiotypic network theory, idiotype’s complementary structures can be found not only on antigens but also on antibodies of different idiotypes**. The immune system after producing specific antibodies to an antigen (Ab1), continues to produce antibodies to the Ab1 idiotopes (Ab2). The particular Ab2 which fit into the antigen binding site of the Ab1, can induce a specific immune responses against the nominal antigen. Thus, the idiotopes could be used as surrogates of any existing antigen. Immunization with Ab2 can lead to the generation of anti-anti-Id antibodies (Ab3) that recognize the corresponding original antigen identified by the Ab1. Several Ab2 have been used to induce specific and protective immunity against tumor antigens.

The idiotypic network hypothesis predicts that due to the huge diversity of immunoglobulin variable regions, and since each antibody will bind its nominal antigen and also other immunoglobulins, within the immune network the universe of external antigens is mimicked by idiotypes. According to this concept, immunization with a given antigen will generate the production of antibodies against this antigen termed Ab1. This Ab1 can generate a series of anti-Id antibodies against Ab1 termed Ab2. The particular anti-Ids which fit into the antigen binding site of the Ab1, can induce a specific immune responses against the nominal antigen. Then, a practical consequence of the idiotypic network theory was that the idiotopes could be used to mimic any existing antigen and used as surrogate antigens. Immunization with Ab2 can lead to the generation of anti-anti-Id antibodies (Ab3) that recognize the corresponding original antigen identified by the Ab1 (**Figure 1**). Several such Ab2 have been used to trigger the immune system to induce specific and protective immunity against tumor antigens (Miller et al., 1982; Jerne, 1985; Lee et al., 1985; Raychaudhuri et al., 1986).

Although Jerne, in his original network hypothesis, and later Coutinho with the second generation networks outlined the importance of naturally occurring idiotypic complementaries, must of the studies using anti-Id as vaccines are focus on the great mimetic capacity of idiotypes, no in activating network related properties, like immune regulation and natural immune surveillance. Beyond the functional mimetic capacity, those anti-Id antibodies related with antigens connected and regulated though networks due to their importance for organisms’ homeostasis, could be able to activate natural antibodies secreting B cells. Their antigens, especially self-antigens, could be the suited ones to get targeted through the idiotypic vaccination. This could be the case of gangliosides.

## ANTI-GANGLIOSIDE IDIOTYPIC VACCINES

Anti-idiotype antibodies that mimic ganglioside have been utilized as active specific immunotherapy in patients with different tumors. Chapman and Houghton (1991) generated in syngeneic mice the anti-Id antibody BEC2 against the anti-GD3 mAb R24. In studies in rabbits this antibody demonstrated its ability to mimic GD3, inducing a specific antibody response of IgG and IgM isotypes. In clinical trials in melanoma patients treated by surgery and with high risk of recurrence (McCaffery et al., 1996) and in patients with SCLC with limited disease (Grant et al., 1999) BEC2 demonstrated to be immunogenic and to induce anti-anti-Id response when administered with the adjuvant BCG. However, it induced specific anti-GD3 antibodies only in a low percentage of patients (Chapman and Houghton, 1991; McCaffery et al., 1996; Grant et al., 1999). The conjugation of BEC 2 to KLH did not increase but reduced the magnitude and frequency of the anti-GD3 response. When anti-GD3 antibodies were induced, they were detected only by ELISA, not by TLC immunostaining (Ritter et al., 1991) or by flow cytometry against GD3-positive melanoma cell lines, suggesting that these anti-GD3 antibodies had a relatively low avidity for cell surface GD3. A phase III trial with 515 patients with limited-disease SCLC after a major response to chemotherapy and chest radiation was performed with BEC2/BCG. This trial failed to show any survival advantage for vaccinated patients. Only one-third of the patients elicited an anti-GD3 response. Among vaccinated patients, a trend toward prolonged survival was observed in those who developed the humoral response (*P* = 0.085), so that it was suggested that the induction of higher titers of antibodies in a larger proportion of patients could make an impact on median survival (Giaccone et al., 2005).

Another trial that targeted a ganglioside, utilized a vaccine composed of an anti-Id mimicking GD2 injected with the adjuvant QS21, a preparation called TriGem. The anti-Id mAb, called 1A7 is a functional mimic of a specific epitope in the ganglioside GD2. In preclinical studies in mice, rabbits, and monkeys the immunization with 1A7 antibody induced a specific IgG response against the ganglioside, capable of causing the lysis of GD2-positive cells on ADCC assays (Sen et al., 1998).

Foon et al. (1998) initiated a clinical trial in patients with advanced melanoma, which were given anti-Id mAb 1A7 with the adjuvant QS21. All sera showed an anti-anti-Id response mainly of the IgG1 isotype. The purified Ab3 from all patients inhibited the binding of the Ab1 to a GD2-positive cell line and to purified GD2. In addition, sera specifically reacted with tumor cells expressing GD2 and were positive in ADCC studies. One patient had a complete clinical response and 6 patients, of a total of 12 enrolled in the trial were stable from 9 to 23 months. In a similar trial, 47 patients with advanced melanoma received 1, 2, 4, or 8 mg doses of TriGem. Hyperimmune sera from 40 of the 47 patients showed an anti-anti-Id response of IgG isotype that specifically bound purified GD2. One patient had a complete response that persisted at 24 months, and 12 patients were stable from 14 to 37 months (median, 18 months). These results showed that this vaccine had minimal toxicity, induced a strong response against GD2 and seemed to have a favorable impact on the reduc-tion of disease progression and survival of patients (Foon et al., 1998, 2000).

In 2003, Basak and colleagues generated Ab2 against the anti-GD2 mAb ME361. These Ab2s induced a specific DTH response in mice against melanoma cell lines that express this ganglioside. Furthermore, these antibodies were able to induce proliferative responses in cells from a melanoma patient confronted with human melanoma cells expressing GD2 *in vitro*, demonstrating the ability of these antibodies to induce cellular responses against carbohydrate antigens (Basak et al., 2003).

Several evidences have shown that tumor antigen-specific antibodies Ab1, used in preclinical experiments or for diagnostic and/or therapeutic purposes, may contribute to anti-tumor effects by triggering the idiotypic cascade and inducing a tumor antigen-specific immune response. The triggering of the idiotypic cascade has been reported to be associated with a favorable clinical response to antibody-based therapy in patients with neuroblastoma, colorectal carcinoma, ovarian carcinoma, and non-Hodgkin lymphoma (Koprowski et al., 1984; Saleh et al., 1993; Cheung et al., 1994, 2000; Schultes et al., 1998). GD2 ganglioside-specific antibodies have been induced in patients with neuroblastoma treated with anti-GD2 ganglioside antibodies.

Treatment with the anti-GD2 monoclonal antibody 3F8 (Ab1) at the time of remission prolonged the survival of children with stage 4 neuroblastoma. Among 34 patients treated with this antibody at the end of chemotherapy 14 were alive, and 13 (1.8–7.4 years at diagnosis) were progression-free (53–143 months from the initiation of 3F8 treatment) without further systemic therapy at the moment of the report. This long-term progression-free survival and survival correlated significantly with the induction of Ab3 anti-GD2 response (Cheung et al., 2000). These results reinforce the importance of GD2 as a tumor target, and the connectivity capacity of anti-ganglioside antibodies.

Our group has developed a vaccine preparation featuring a murine anti-Id mAb related to the NeuGc-containing ganglioside antigen model. This Ab2, named 1E10 (Vázquez et al., 1998), was generated from the immunization of BALB/c mice with P3, an idiotypic antibody (Ab1) that recognizes NeuGc-containing gangliosides, sulfated glycolipids, and antigens present in different human tumors including those from the lung. This Ab1 is highly immunogenic in the syngeneic model, inducing an anti-Id response in the absence of adjuvant or carrier protein. Furthermore, the Ab1 P3 was able to activate NeuGcGM3 related idiotypic cascade, since antibodies against NeuGcGM3 (Ab3, Ab1 like) were detected in chickens immunized with this Ab1. The detection of Abs with this specificity in animals immunized with an Ab1 suggested that the elicited Ab2s behaved as a ganglioside surrogate inducing a specific Ab3 response against this antigen.

**FIGURE 2:**
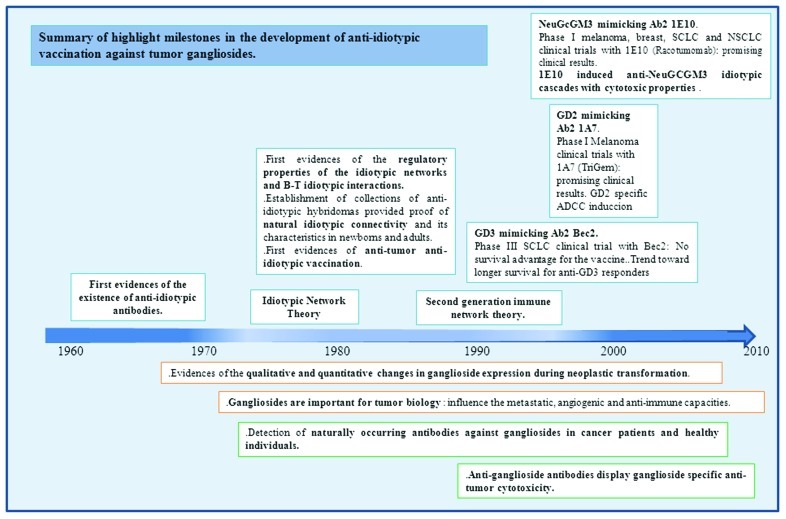
**Timeline of highlight milestones in the development of anti-idiotypic vaccination against tumor gangliosides**.

Preclinical data published by our group suggest that P3 and 1E10 mAb could be able to activate idiotypic networks, involving both B and T cells. Lymph node cells from BALB/c mice immunized with P3 mAb proliferated *in vitro*, in a dose-dependent manner, not only in response to P3 mAb but also to 1E10 mAb, suggesting the existence of a naturally occurring B/T cell idiotypic network (Perez et al., 2002). Phase I clinical trials have proven the safety and immunogenicity of 1E10 Id vaccination in melanoma, breast, and lung cancer patients (Alfonso et al., 2002, 2007; Diaz et al., 2003; Neninger et al., 2007; Hernandez et al., 2008). In all the cases, 1E10 idiotype proved to be immunodominant, since the induced anti-anti-Id response was significantly higher than the anti-isotypic response. Similar results were obtained when monkeys and chickens were immunized with 1E10 mAb (Hernandez et al., 2005), suggesting that 1E10 mAb Id immunodominance is not a species-depending property. High titer antibody responses to NeuGc-containing gangliosides were measured in the sera of cancer patients and were confirmed by TLC immunostaining. Interestingly, a fraction of non-suppressible anti-NeuGc-containing ganglioside Abs was demonstrated after the adsorption of the patients’ sera with 1E10 mAb, suggesting that 1E10 Id vaccination was activating natural anti-NeuGcGM3 responses (Hernandez et al., 2008). The antibodies that recognize both 1E10 and the ganglioside (Id^+^Ag^+^) and the ones that recognize the ganglioside but not the immunizing Ab3 (Id^-^Ag^+^), recognized and induced the death of tumor cells expressing NeuGcGM3 by an oncotic necrosis mechanism. Those patients who developed IgG and/or IgM Abs against NeuGcGM3 showed a longer survival time. We hypothesize that 1E10 Id vaccination could be activating an existing idiotypic cascade related with *N*-glycolylated gangliosides, which would amplify the antigen-specific immune response to a tumor-associated neo-self antigens. This therapeutic concept goes beyond the classical concept of antigen mimicry. A randomized, double blind phase II clinical trial is ongoing to evaluate the clinical effect of 1E10 mAb vaccine in NSCLC patients and to define the value of the Abs induced by the anti-Id treatment as real predictors of clinical outcome. For a chronological representation of the principal milestones in the development of anti-Id vaccines against tumor expressed gangliosides see **Figure 2**.

## CONCLUDING REMARKS

At present, most of the anti-Id vaccine approaches are based and study the mimetic capacity of the anti-Id antibodies, without searching for their immunoregulatory or natural anti-tumor potential. The use of anti-Id antibodies as immunogens could offer the possibility not only to generate Ab3 antibodies against their own idiotopes, but also to inducing a cascade of Id–anti-Id interactions leading to an amplified and long lasting immune response against the nominal antigen. This immunization could also involve T cells in the response against glycolipidic antigens. The expansion of natural antibodies repertoire by idiotypic vaccination could even participate in the lysis of tumor cells by the activation of evolutionarily fixed anti-tumor mechanisms. A naturally occurring antibody response against ganglioside, which has shown to carry anti-tumor properties, exists in healthy individuals and cancer patients. The idiotypic vaccination could be an optimum way to activate the idiotypic B and T cell cascades involving the natural responses against these antigens.

## Conflict of Interest Statement

Dr. Ana M. H. Vázquez is an inventor of patents related with P3 mAb and its anti-idiotypes, however, she has signed the assignment of her rights to the assignee Center of Molecular Immunology. The other authors have no conflicts to report.
